# Multi-layered model for simulating the *in vivo* terahertz response of human skin

**DOI:** 10.1364/BOE.579637

**Published:** 2025-11-18

**Authors:** Benjamin G. Page, Jacob J. Young, Agrima Agarwal, Xiangyu Guo, Joseph Hardwicke, Emma Pickwell-MacPherson

**Affiliations:** 1 Department of Physics, University of Warwick, Coventry, CV4 7AL, UK; 2 Institute of Applied & Translational Technologies in Surgery (IATTS), University Hospital Coventry and Warwickshire, Coventry, CV2 2DX, UK; 3 University of Warwick, Medical School, Coventry, CV4 7AL, UK

## Abstract

Quantitative methods of evaluating the state of skin are highly beneficial for both diagnosis and treatment monitoring. The development of such methods relies on understanding how changes in skin properties affect the quantitative response. Effective modelling is often a crucial step in building this understanding. This work introduces a multi-layered model for simulating the *in vivo* terahertz response of skin, demonstrating how variations in skin properties may alter the measured signal. Furthermore, we hypothesise that the observed attenuation in the terahertz signal during an *in vivo* measurement is primarily a result of skin deformation and flattening under compression by the imaging window. Finally, we fit our model to measured data and extract optimised values for a skin deformation parameter.

## 1. Introduction

The ability to quantitatively assess the state of human skin is of great utility for those looking to develop treatment or diagnosis tools for dermatological conditions. Indeed, the incidence rates of both melanoma and non-melanoma skin cancers are on the rise in the UK [1,2], and psoriasis and eczema continue to affect a sizeable proportion of the population (2% and 5-10%, respectively) [3,4]. Terahertz (THz) light has shown promise for *in vivo* sensing of skin cancer patients [5], along with a plethora of other biomedical applications, including corneal hydration assessment [6] and burn wound monitoring [7]. The field is continuing to grow, with recent advances in biomedical THz technologies focussing on fast acquisition rates, for example through the use of ASynchronous OPtical Sampling (ASOPS) or Electronically Controlled OPtical Sampling (ECOPS) [8,9]. However, these systems are often expensive and come at the cost of signal-to-noise. The applicability of THz for biomedical use lies in its sensitivity to the hydrogen bonds present within liquid water, providing a unique modality of probing biological water content. Furthermore, unlike other imaging techniques such as X-rays, THz light is non-ionising, making it suitable for *in vivo* measurements which may require prolonged THz exposure.

Improvements in techniques such as THz sensing often rely on accurate and efficacious modelling to inform data analysis approaches and explore possible contrast sources. Such models have been previously used to investigate the effects of pressure on terahertz skin reflectivity and to calculate hydration increases in skin [10–12].

### 1.1. Skin occlusion

The nature of *in vivo* studies and the dynamic processes that occur in biological systems mean measurements are often accompanied by a unique set of considerations and challenges. For example, the high-water sensitivity of THz is both a useful property for hydration sensing and a limitation for signal propagation and penetration. On this premise, we introduce the dynamic process of occlusion, which refers to the blocking of skin by an impermeable barrier. Once blocked, water loss through the skin’s surface is prevented and water accumulates within the outermost layers (the stratum corneum), increasing the hydration [13]. This phenomenon is frequently utilised in maximising the percutaneous absorption of various pharmaceuticals by artificially modifying the skin’s hydration gradient [14]. However, the effects of occlusion must also be considered for *in vivo* skin measurements since reflection THz probes usually have an impermeable imaging window (e.g. quartz) at the tip to contact the skin.

**Fig. 1.**
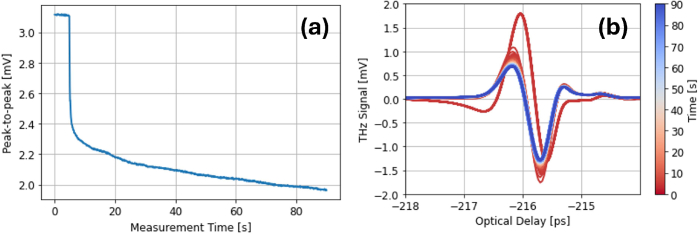
(a) The ’occlusion curve’ showing the decrease in signal peak-to-peak. (b) THz signal attenuation.

Cole *et al.* originally proposed that hydration increase due to occlusion by the imaging window was the reason for an observed decrease in the THz signal peak-to-peak over 15 minutes [15]. The attenuation of the THz signal as a function of measurement time has been dubbed the ’occlusion curve’, as shown in Fig. 1. This idea has since been developed, with Ding *et al.* using the stratified media model (SMM) to estimate that stratum corneum (SC) hydration increases from 20% to 30% within a 60-second measurement using a quartz window at an applied pressure of between 0.6-1.1 *Ncm*^−2^ [10,12].

However, literature suggests that the increase in hydration due to occlusion occurs over a longer time scale than 60 seconds. Faergemann *et al.* found that skin water content increased by around 8% after 8 days of occlusion with a polymer plastic film [16]. Anderson *et al.* found a similar percentage change in water content after 24 hours of occlusion [17]. Whilst there is some evidence that suggests that SC hydration can increase even after shorter-term occlusion (30 minutes), there is currently no evidence for a large-scale hydration increase over 1 minute. Furthermore, it has been shown that the water content of the skin may temporarily decrease during compression due to interstitial water displacement [18].

As such, this work considers an alternative mechanism that may drive THz signal attenuation over shorter time scales. In doing so, we develop a multi-layered model to accurately simulate the *in vivo* terahertz response of skin. We parameterise changes to the skin state and fit our model to measured data to quantitatively assess the change in skin state as a function of measurement time. From here on, we shall refer to the ’occlusion curve’ as the signal attenuation curve to avoid confusing the reader about its exact origin.

### 1.2. Skin deformation

**Fig. 2.**
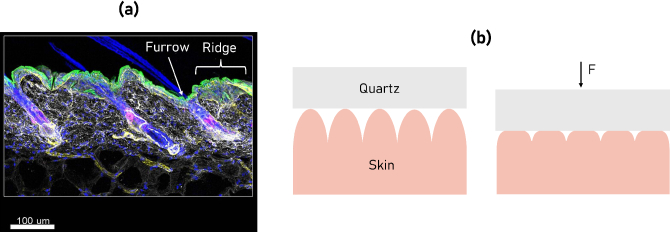
(a) Cross-sectional image of back skin showing the furrows and ridges. Licensed under CC BY-SA 3.0. from National Institute of Allergy and Infectious Diseases. (b) Skin ridge flattening due to compression of force *F* by a quartz window.

**Fig. 3.**
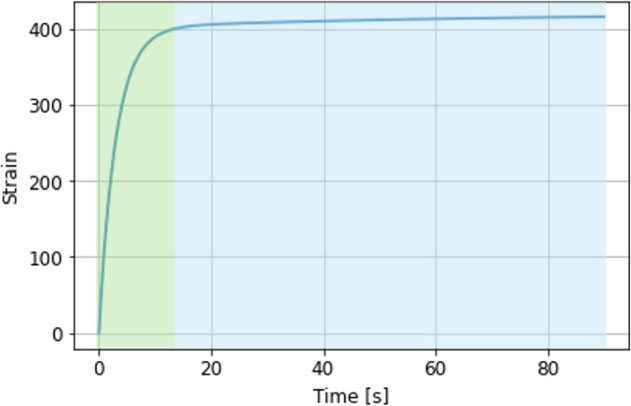
A typical creep deformation curve for skin showing two phases of deformation: fast, elastic-dominated (shaded in green), and slow, viscous-dominated (shaded in blue). For illustration purposes only, not-to-scale.

We propose that the signal attenuation observed during a 60 second measurement using an imaging window is primarily a result of the deformability and compressibility of the skin. The topography of the skin is formed of various ridges, furrows, and large-scale wrinkles [19]. Initially, only the crest of the ridge will contact the window; however, during compression by an imaging window, the ridges deform, causing an increased amount of the skin’s surface area to contact the imaging window [20]. This is schematically shown in Fig. 2. Continual compression leads to an increasing amount of deformation and may alter the plasticity of the skin, making it easier for further deformation to occur. This effect is related to the viscoelasticity of the skin, whereby the skin exhibits both viscous and elastic behaviour under stress [21]. This is displayed in the creep deformation response of the skin under constant stress, which is shown in Fig. 3. The two phases of creep deformation are an initial fast, elastic-dominated exponential increase in strain, followed by a slower, viscous-dominated linear increase [22].

We may understand the signal attenuation curve in the context of viscoelastic deformation of skin. To begin with, the skin undergoes fast elastic deformation, and the surface area of skin contacting the quartz window increases accordingly, leading to a sharp decrease in the THz signal peak-to-peak (P2P). Further deformation occurs on a slower time-scale, causing an increasing amount of skin to flatten against the quartz. In this region, the THz signal P2P decreases quasi-linearly.

To investigate this hypothesis, we developed a model to simulate the effect of skin deformation on the *in vivo* THz signal.

## 2. Modelling

**Fig. 4.**
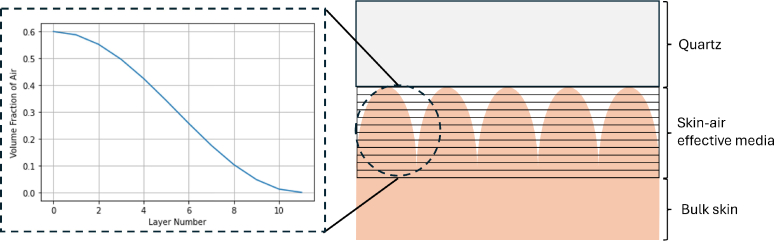
A schematic of the multi-layered model including a quartz layer, a 12-layer effective medium of skin and air, and a bottom bulk skin layer.

Our model consists of a multi-layered system of skin-air effective media, situated above a homogeneous bulk skin layer. The model is shown schematically in Fig. 4. To represent the skin’s furrows and ridges, we introduce a number of ridge layers. Each of these layers is treated as a two-component effective medium, composed of skin and air, where the volume fraction of air varies according to a squared cosine function, intended to approximate the typical profile of a skin ridge. As we move downward through the ridge layers, the air volume fraction progressively decreases. The effective dielectric properties of the N^*th*^ ridge layer are calculated using the Landau–Lifshitz–Looyenga (LLL) effective medium model, given by:

(1) $$\varepsilon_{\text{effective}}^{N}={\left(\left(1-V_{A}^{N}\right)\cdot \varepsilon_{\text{skin}}^{1/3}+V_{A}^{N}\cdot \varepsilon_{\text{air}}^{1/3}\right)}^{3}$$

where $\varepsilon_{\text{effective}}^{N}$ is the effective dielectric permittivity of the N*^th^* layer, $V_{A}^{N}$ is the volume fraction of air in the N*^th^* layer, and $\varepsilon_{\text{skin}}$ and $\varepsilon_{\text{air}}$ are the dielectric permittivities of *in vivo* skin and air, respectively. The LLL effective medium model was chosen as it accounts for arbitrarily shaped inclusions, unlike other models which impose a sphericity criterion. Furthermore, it has previously been shown that the LLL model is the most well-suited to modelling biological tissue [23,24].

Considering the other layers, quartz has a refractive index of 1.98 and a thickness of 2mm, and bulk skin has a frequency-dependent refractive index determined from experiment. The thickness of each ridge layer (df) is equal, and set between 2 and 10 *μ*m. Thus, for six ridge layers, the total ridge height is 12-60 *μ*m which is consistent with measured values for the furrow depth of the forearm [25].

To determine the optimum number of ridge layers, we performed a robustness analysis by calculating the mean and standard deviation of the simulated error over a range of total ridge heights, *d*, and $V_{A}^{1}$ values. The process is as follows: we define an error matrix of size [M,N] where *M* is the number of $V_{A}^{1}$ values (for example, 10 spanning 0, 1) and *N* is the total number of ridge layers, *L* (for example 15, spanning 1, 15). For a chosen total ridge height, *d*, we simulate the terahertz signal for all combinations of $V_{A}^{1}$ and *L*. We calculate the error as the squared L2 norm of the difference between the simulated and measured signal.

**Fig. 5.**
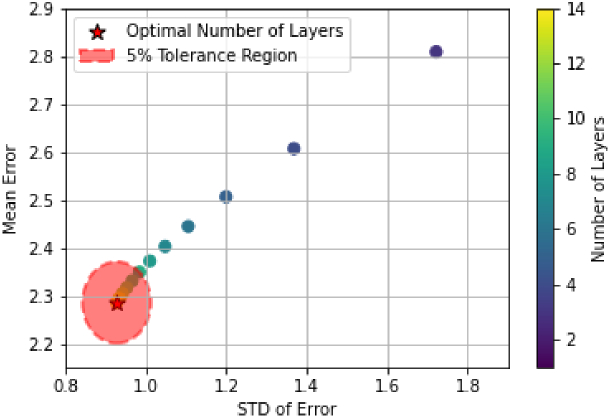
Identifying the optimum number of ridge layers based on a robustness metric calculated from the combined mean and standard deviation (STD) of the error. The 5% tolerance limit is shown by the shaded region. The theoretical optimal number of layers would exist at (0, 0).

A robustness metric was calculated by combining the mean and standard deviation of the error across all $V_{A}^{1}$ values for each *L*. The optimum number of ridge layers at a chosen *d* is selected by minimising the robustness metric. We also identify a set of acceptable *L* values that score within 5% of the minimum metric value. An example of this process for a total ridge height of 30 *μ*m is shown in Fig. 5. Using this process, for total ridge heights between 15 and 60 *μ*m, it was found that L=12 is the lowest value that appears in all acceptable sets, and thus was selected as the optimum number of ridge layers.

**Fig. 6.**
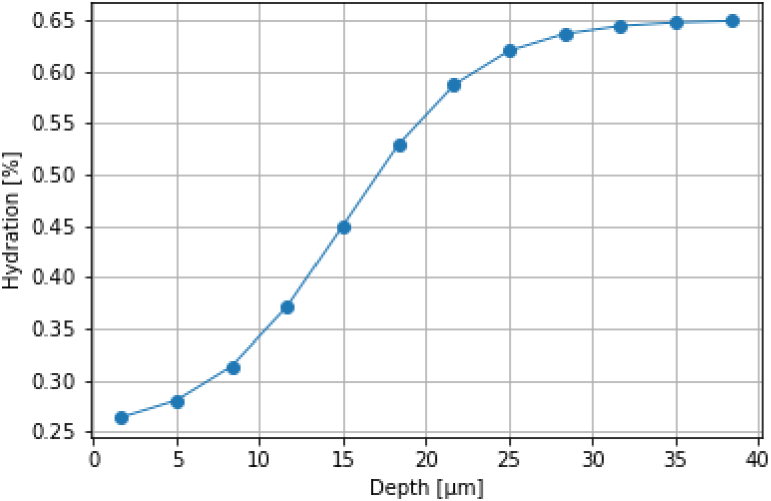
The hydration profile as a function of depth into the ridge layers, increasing from 20% at the surface to 65% at the bottom of the final layer.

We also considered the hydration profile through the ridge layers; this has a direct impact on the dielectric properties of each layer and will thus affect the simulated THz signal. In our model, skin is considered an effective medium of ’dry’ biological background and bound water. It has previously been shown using Raman spectroscopy that there is a water concentration gradient across the skin’s layers, with the hydration increasing from approximately 30% at the surface to 60% at 35 *μ*m depth [26]. To integrate this effect, as shown in Fig. 6, we have used Raman spectroscopy data to estimate the hydration of each ridge layer - the centre point of each layer is shown by the circle.

Once the dielectric permittivities of each of the model’s layers have been calculated or defined, a recursive Fresnel model (Eq. (2)) is used to calculate the total effective reflection coefficient of the system:

(2) $$r_{i}=\frac{r_{i,i+1}+r_{i+1}e^{2j\delta_{i+1}}}{1+r_{i,i+1}r_{i+1}e^{2j\delta_{i+1}}}$$

(3) $$\delta_{i+1}=\frac{2\pi }{\lambda }n_{i}d_{i}\cos\theta_{i}$$

where $r_{i}$ is the overall reflection coefficient at the i*^th^* layer and $\delta_{i+1}$ is the phase thickness of the i*^th^* + 1 layer. $r_{i,i+1}$ is the Fresnel reflection coefficient between the i*^th^* and i+1*^th^* layer, $n_{i}$ and $d_{i}$ are the refractive index and thickness of the i*^th^* layer, respectively [27].

**Fig. 7.**
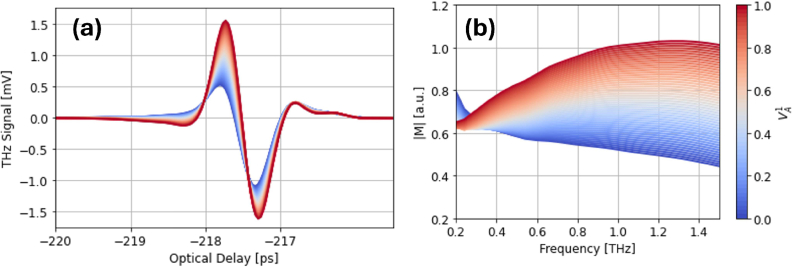
The effect of $V_{A}^{1}$ on the simulated terahertz response in (a) the time-domain and (b) the frequency-domain.

In our model, skin deformation and flattening are parameterised by the volume fraction of air in the first ridge layer, $V_{A}^{1}$ . A decrease in $V_{A}^{1}$ corresponds to skin flattening against the quartz window; thus, perfectly flattened skin would have $V_{A}^{1}$ equal to zero. The effect of $V_{A}^{1}$ on the simulated THz response is shown in Fig. 7. Decreasing $V_{A}^{1}$ from 1 to 0 (red to blue) causes clear attenuation in the signal, which mimics the attenuation seen *in vivo*.

**Fig. 8.**
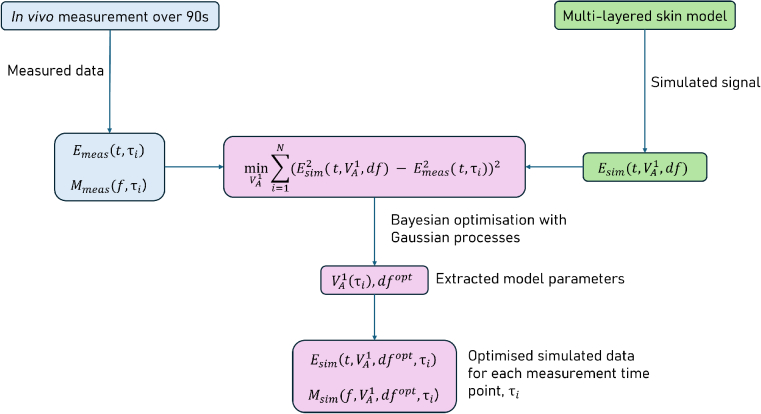
The optimisation process used to extract $V_{A}^{1}$ values as a function of measurement time, *τ*.

To extract values for $V_{A}^{1}$ , and to investigate the model’s efficacy, we fit it to *in vivo* measurements taken from the volar forearm. Fitting was done via a loss function minimisation of the form:

(4) $$\min_{V_{A}^{1}}\sum_{i=1}^{N}{\left(E_{\text{sim}}^{2}(t,\tau_{i},V_{A}^{1},df^{\text{opt}})-E_{\text{meas}}^{2}(t,\tau_{i})\right)}^{2}$$

where $df^{\text{opt}}$ is the optimised value of ridge layer thickness, $E_{\text{sim}}(t,\tau_{i})$ is the simulated time-domain THz signal at a given time point during compression, $\tau_{i}$ , and $E_{\text{meas}}(t,\tau_{i})$ is the measured THz time-domain signal at the aforementioned point. $df^{\text{opt}}$ is found by minimisation of a similar loss function, where $V_{A}^{1}$ is fixed at 0.5. Minimisation is robust and achieved using Bayesian optimisation with Gaussian processes. The searching bounds for $V_{A}^{1}$ are [0, 1] and [2, 10] *μ*m for df . The optimisation process is shown in Fig. 8.

**Fig. 9.**
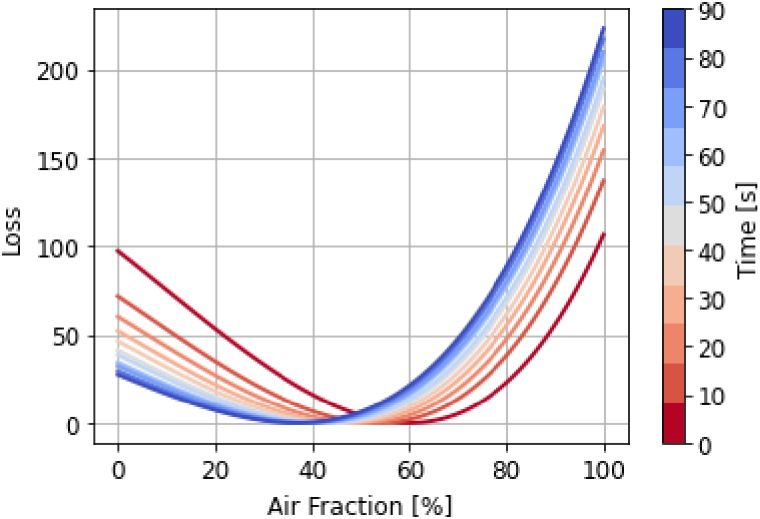
The loss surface of the model at each index of the fitting process between 0 and 90s, showing that the $V_{A}^{1}$ (air fraction) parameter is identifiable and varies with measurement time.

After fitting, we can explore the loss surface of the model by plotting the objective function loss as a function of $V_{A}^{1}$ . As is shown in Fig. 9, the loss surface is convex with a clear minimum value. Hence, there is an optimal value of $V_{A}^{1}$ that the minimisation process converges towards during fitting. It is also clear that this minimum value shifts with measurement time; $V_{A}^{1}$ is an identifiable and time-varying parameter.

## 3. Methodology

Measured data was obtained *in vivo* from the volar forearm of a patient in a clinical setting as part of the TERABOTICS study at University Hospitals Coventry and Warwickshire (UHCW). Ethical approval was awarded by the Health Research Authority (HRA) and Health and Care Research Wales (HCRW) for The TERABOTICS study (IRAS project ID: 270335). Appropriate informed consent was taken from each human participant before measurements. All research was performed in accordance with the relevant guidelines and regulations, including in accordance with the Declaration of Helsinki.

Our in-house robotic terahertz spectrometry setup, the PicoBot, was used to perform all measurements - details of which can be found in [5,28]. The contact force was maintained at 5N for the duration of the single-point measurement. The acquisition rate was around 12Hz - 1093 pulses were recorded during a 90s measurement.

## 4. Results and discussion

### 4.1. Roughness testing

**Fig. 10.**
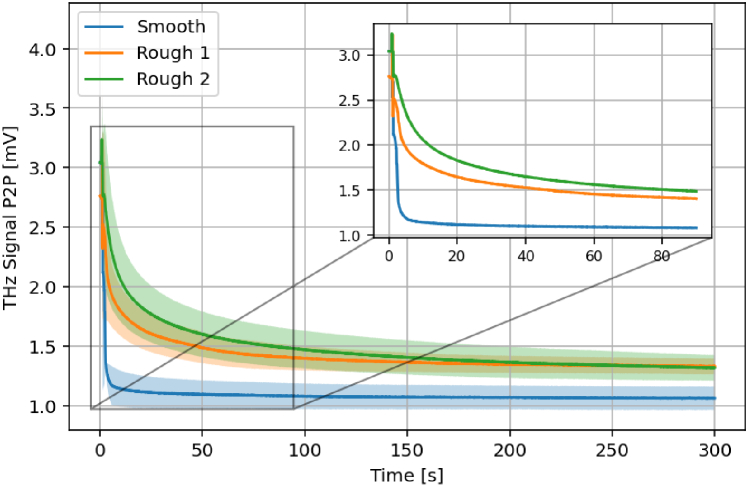
The effect of surface roughness on the mean and standard deviation of the THz signal peak-to-peak as a function of measurement time over 5 minutes of compression with 3N of force. The inset shows the signal attenuation over 90s.

To study the effect of surface roughness on the THz response and to investigate its role in signal attenuation, we have used Play-Doh as a skin-mimicking phantom. Play-Doh, like skin, is viscoelastic, and its THz refractive index is similar to that measured for skin. Hence, the effect of surface roughness deformation on the THz response in Play-Doh should provide insight for *in vivo* skin measurements. Samples of Play-Doh were imprinted with different surface textures: smooth (no imprinted texture), rough 1 (100-grit sandpaper, Ra≈30μm [29]) and rough 2 (60-grit sandpaper, Ra≈60μm [29]). Here, Ra represents the arithmetic mean of height deviations from the mean value. For each surface texture, we have measured the THz response of the sample over 5 minutes with 3N of force applied over the total area of the quartz imaging window, 380 mm^2^, in the z-direction. Each measurement has been repeated three times. A comparison between the mean signal attenuation curves for each texture is shown in Fig. 10.

For each texture, there is a clear exponential decay in the THz signal peak-to-peak, similar to that for an *in vivo* skin measurement. However, the time constant of the exponential appears to be roughness-dependent. Greater values of surface roughness are correlated with an increase in the exponential time constant i.e., it takes longer for the THz signal P2P to reach a steady-state. There also appears to be a link between the steady-state peak-to-peak $\left(P2P_{ss}\right)$ and the surface roughness. For the smooth sample, $P2P_{ss}$ is significantly lower than for either of the rough samples. We hypothesise that $P2P_{ss}$ is dependent on the total surface area of the sample that is capable of contacting the quartz window. For a smooth sample, a maximal amount of surface area is available for contact; however, for a rough sample, there is a limit imposed on the maximum surface area set by the surface roughness. During the measurement, the contact pressure from the quartz window causes the rough surface to smooth out - this is seen in the tail of the exponential. The slower, quasi-linear decrease in P2P after the initial sharp drop is explained by the viscoelasticity of the Play-Doh amount of surface deformation is required to achieve maximal contact between the sample surface area and the quartz. For the two rough cases, owing to the surface roughness, more deformation is required before maximal contact is reached. These results suggest that the shape of the *in vivo* THz signal attenuation curves are dependent on the roughness and deformability of the skin. Hydration changes due to occlusion may have an effect at longer timescales, but over short scales (for example, 90 seconds), surface feature deformation and compression are likely the dominant effects driving signal attenuation.

### 4.2. Pre-occluded skin

**Fig. 11.**
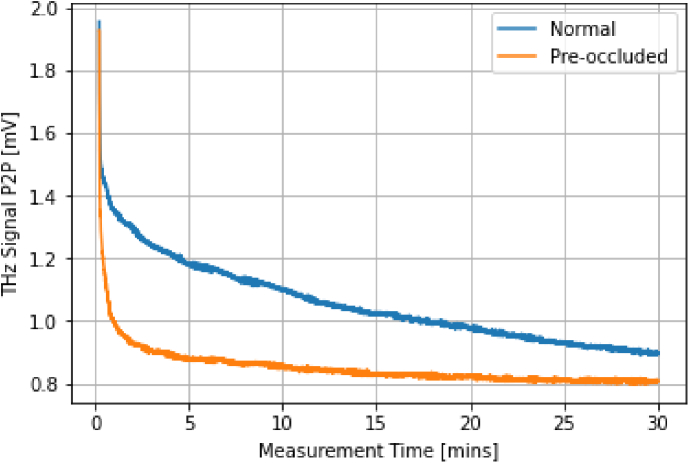
The signal attenuation curves obtained from a volunteer’s volar forearm, highlighting the effect of pre-occlusion on the measurement.

To investigate the effect of occlusion on the *in vivo* THz response, two signal attenuation curves were measured over 30 minutes - this is shown in Fig. 11. Both measurements were of a volunteer’s volar forearm; one had been pre-occluded by wrapping the forearm in plastic for 1 hour, and the other had not been pre-occluded (labelled ’normal’). For the pre-occluded case, the exponential decay is much faster and a steady-state peak-to-peak is reached around 20 minutes. In the normal case, the attenuation continues over the entirety of the 30 minute measurement, and no steady-state is reached. The normal curve also appears to converge towards the pre-occluded steady-state, suggesting that the pre-occluded case has achieved a maximal impedance.

This result can also be understood in the context of surface feature deformation and skin flattening. For the pre-occluded case, the hydration of the skin is increased, leading to an increase in its compressibility and plasticity. It follows that skin flattening may occur at an amplified rate, as shown by the faster exponential decay. After some time, the skin is maximally flattened, and a steady state is achieved. Of course, in this case, we expect the hydration of the skin to further increase the impedance compared to the normal case. Thus, the minimum P2P reached for pre-occluded skin is a result of increased skin flattening and hydration. In the normal case, skin flattening occurs at a slower rate and is not maximally flattened after 30 minutes. Furthermore, the hydration of the skin is likely lower than for pre-occluded skin and hence may increase during the measurement. Both effects contribute to the exponential decay in the signal P2P, but neither reaches a maximal steady-state during 30 minutes.

### 4.3. Comparison to measured data

With the effect of roughness and compression in mind, we now discuss the results of fitting our model to measured *in vivo* data. In doing so, we extract the optimised ridge layer thickness, $df^{\text{opt}}$ , and time-dependent values for the volume fraction of air in the first ridge layer, $V_{A}^{1}$ . Consequently, we accurately replicate THz signal attenuation in both the time and frequency domains.

**Fig. 12.**
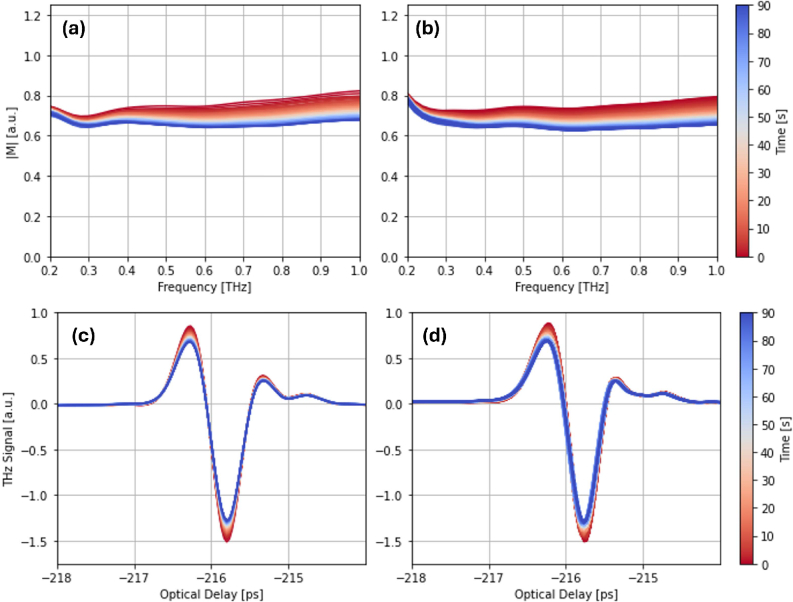
A comparison between the signal attenuation in the frequency and time-domain for both the measured and simulated waveforms. (a, c) The simulated data. (b, d) The measured data.

**Fig. 13.**
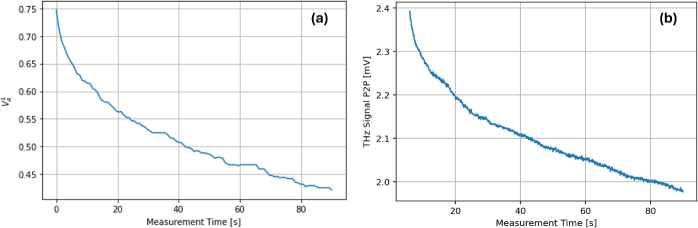
A comparison between the (a) extracted values of $V_{A}^{1}$ and (b) the signal attenuation curve.

Figure 13(b) shows the measured terahertz signal attenuation in the time-domain; the exponential decay of the peak-to-peak amplitude is clear and drops by 0.41 mV over 90s. The changes to the time-domain terahertz pulse are also clear; there are observable decreases in the primary and secondary peak and in the primary trough - see Fig. 12(d). It is important to note that some of these changes are observable only in the ’raw’ THz signal. Previous works have focussed on changes in the impulse response function as discussed in [30]. Whilst this provides a more intuitive framework for direct comparison between pulses, it suffers from a significant over-smoothing problem and suppresses useful signal information [31]. As such, we choose to consider attenuation of the raw terahertz signal, which we find more robust and reliable. In the frequency-domain, we consider attenuation of the deconvolved response, *M*, as is typical for such analyses - see [31]. Again, it is important to note that we have not applied any frequency filters to this data, as it has been shown to over-smooth the signal even in the high signal-to-noise ratio region (between 0.2-1.5 THz).

Figures 12(c) and (d) compare the measured and simulated THz signal in the time-domain. Model fitting is done at every 10th point across the measurement duration, and at each of these points, the root-mean-square error between the simulated and measured THz pulse has been calculated. Visually, the simulated and measured time-domain pulses show a high level of similarity. This is also seen in the frequency-domain; signal attenuation is accurately replicated by a decrease in $V_{A}^{1}$ over the measurement period. As is clear from Fig. 13, the $V_{A}^{1}$ parameter is highly correlated with the signal attenuation curve. Over the 90s measurement, the total change, $\Delta V_{A}^{1}(0\to 90s)$ , is 0.19 (0.57 to 0.38), which corresponds to a simulated decrease in the signal P2P of 0.38mV.

**Fig. 14.**
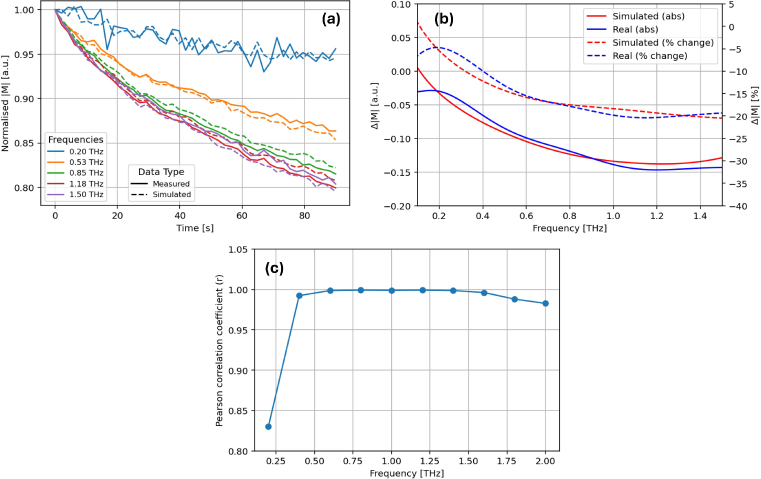
(a) The attenuation of the simulated (dashed) and measured (solid) magnitude of the frequency response, M, over 90s. (b) A comparison between the frequency-dependence of the simulated and measured signals throughout the 90s measurement, quantifying the total change in the frequency-response. (c) The Pearson correlation coefficient of the frequency-component attenuation.

Considering the frequency-domain in more detail, Figs. 14(a) and (b) show how different frequency components are attenuated over the measurement period. From Fig. 14(a), we can see a strong correlation between the simulated and measured signals for all frequencies. The Pearson correlation coefficient (*r*) for each frequency is listed in the legend. Figure 14(b) shows the absolute and percentage change in the magnitude of the frequency response, M, from the first to the last point in the measurement between 0.2 and 1.5THz. Both the real and simulated data show the same pattern where lower frequencies (0.2-0.5THz) are more significantly attenuated than the higher frequency (>0.5THz) components. Finally, Fig. 14(c) shows *r* for a range of frequencies between 0.2 and 2THz - it peaks around 0.6THz and remains at this level until 1.5THz, where it begins to drop. As such, it is concluded that our fitting process is robust between 0.3 and 1.5THz, which is comparable to the bandwidth of our system.

## 5. Conclusion and future work

In this work, we develop a multi-layered system for modelling the *in vivo* terahertz response of skin. In doing so, we hypothesise that terahertz signal attenuation observed at the beginning of a contact measurement is primarily a result of skin deformation and flattening by the imaging window. Our model accommodates for this effect by altering the volume fraction of air in each of the ’ridge layers’ - parametrised by $V_{A}^{1}$ . The time-dependence of this deformation parameter is extracted via a fitting to measured data from a patient’s volar forearm. The results of this fitting suggest that $V_{A}^{1}$ accurately and effectively parametrises the observed signal attenuation. The ability to accurately model the terahertz response of biological systems is highly advantageous to aid in our understanding of how changes to the state of the system may affect the terahertz response, and thus be utilised in future diagnostics.

In future work, we aim to investigate the link between the viscoelastic mechanical properties of the skin and the signal attenuation curve. This may provide an understanding of how the shape and properties of the signal attenuation curve could be used as a diagnostic tool in assessing the state of a patient’s skin. We also aim to identify the affect of hydration changes on the signal attenuation curve, and to study the link between hydration and deformability via terahertz attenuation.

## Data Availability

All the data used to produce the plots in this paper are available at [32].
